# For there is nothing either good or bad: a study of the mediating effect of interpretation bias on the association between mindfulness and reduced post-traumatic stress vulnerability

**DOI:** 10.1186/s12888-022-03950-y

**Published:** 2022-05-12

**Authors:** Hannah Deen, Lies Notebaert, Bram Van Bockstaele, Patrick J. F. Clarke, Jemma Todd

**Affiliations:** 1 School of Psychology, University of Sydney, Sydney, Australia; 2 The Matilda Centre for Research in Mental Health and Substance Use, University of Sydney, Sydney, Australia; 3 Centre for the Advancement of Research on Emotion, School of Psychological Science, University of Western Australia, Crawley, Australia; 4 Research Institute of Child Development and Education, University of Amsterdam, Amsterdam, the Netherlands; 5 Affective, Behavioural, and Cognitive Neuroscience Research Group, Curtin University, Perth, Australia

**Keywords:** Mindfulness, Trauma, PTSD, Interpretation bias, Mediation, Mechanism

## Abstract

**Background:**

Despite increasing interest in the association between mindfulness and reduced trauma vulnerability, and the use of mindfulness in the latest interventions for Post-Traumatic Stress Disorder (PTSD), few studies have examined the mechanisms through which mindfulness may influence post-trauma psychopathology. The present study aimed to determine whether negative interpretation bias, the tendency to interpret ambiguous information as negative or threatening rather than positive or safe, mediates the association between higher levels of trait mindfulness and lower levels of PTSD symptoms. Negative interpretation bias was examined due to prior evidence indicating it is associated with being less mindful and post trauma psychopathology.

**Methods:**

The study examined 133 undergraduate students who reported exposure to one or more potentially traumatic events in their lifetime. Participants completed self-report measures of trait mindfulness (Five Facet Mindfulness Questionnaire – Short Form; FFMQ-SF) and PTSD symptoms (Post-Traumatic Stress Disorder Checklist – Civilian version; PCL-C) as well an interpretation bias task that assessed the degree to which participants interpreted a range of everyday hypothetical scenarios to be threatening to their physical and/or psychological wellbeing.

**Results:**

Results of a mediation analysis indicated a significant negative direct effect of trait mindfulness on PTSD symptomatology (*p* < .001). There was no evidence that negative interpretation bias mediated this relationship [BCa CI [-0.04, 0.03)], nor was it associated with trait mindfulness (*p* = .90) and PTSD symptomatology (*p* = .37).

**Conclusions:**

The results of the current study provide further evidence of the link between trait mindfulness and reduced post-trauma psychopathology while providing no support for the role of negative interpretation bias in this relationship.

**Supplementary Information:**

The online version contains supplementary material available at 10.1186/s12888-022-03950-y.

## Introduction

Research suggests upwards of 70% of people will experience a potentially traumatic event in their lifetime, with estimates in some countries reaching as high as 90% [1–3]. While most people who experience a potentially traumatic event will be largely unaffected, it is estimated approximately 9% will develop Post-traumatic Stress Disorder (PTSD) [4]. Many others who do not meet the criteria for PTSD diagnosis will still experience post-traumatic stress symptoms of some kind [4]. PTSD is characterised by the re-experiencing of a traumatic event in one’s mind and body through intrusive flashbacks or nightmares, hypervigilance towards threat cues in one’s internal and external environment, cognitive and behavioural avoidance of threat, and negative changes in cognition and mood following exposure to a potentially traumatic event [4].

Research into how best to prevent and treat PTSD is ongoing and there remains significant room for improving outcomes for people exposed to potentially traumatic events [5]. For example, although rates are highly variable across studies, upwards of 70% of participants in studies examining the most highly recommended evidence-based treatments for PTSD have been shown to retain their PTSD diagnosis following treatment [6, 7]. It has also been estimated that approximately 18% of participants, on average, will drop out of these treatments before completing all components [5].

Recent refinements to psychotherapeutic treatments for PTSD incorporate mindfulness practice as a core component. Mindfulness refers to a mind state that directs attention to the present moment, rather than fixating on thoughts of past and future. It also applies a nonjudgmental and accepting lens to all phenomena that make up the present moment such as the physical environment, thoughts, emotions, and bodily sensations [8]. Research indicates levels of trait mindfulness, that is, a person’s natural capacity to pay attention and maintain attention to the present moment with a nonjudgmental attitude in their daily life, differ in the human population [9]. Research also indicates trait mindfulness is not fixed and that an individual’s capacity for being mindful in daily life can be strengthened through regular mindfulness practice, that is, the practice of actively directing one’s attention to the present moment in an open and nonjudgmental way [9]. Studies have shown that higher levels of trait mindfulness are associated with being more resilient to the psychological effects of trauma [10–14]. Further studies examining mindfulness practice have shown that it can facilitate faster recovery from post-trauma psychopathology [6] and pre-emptively strengthen psychological resilience in populations that are frequently exposed to acute and chronic stress [15, 16].

Despite increasing interest in the association between trait mindfulness and reduced trauma vulnerability, and the use of mindfulness in the latest interventions for PTSD, few studies have examined the mechanisms through which mindfulness may reduce vulnerability to post-trauma psychopathology [12, 17–20]. Understanding the mechanisms of action for any psychological intervention is important for improving its efficacy and efficiency, as this knowledge allows its most essential elements to be identified, refined, and better integrated with other interventions that may affect complimentary mechanisms of change. An appropriate starting point in this line of enquiry is to first identify what mechanisms might explain the apparent natural association between trait mindfulness and reduced vulnerability to the psychological effects of trauma in cross-sectional surveys among the general population prior to conducting more intensive experimental studies that manipulate trait mindfulness through therapeutic intervention.

One candidate mechanism for explaining the association between being naturally more mindful and reduced vulnerability to post-trauma psychopathology not yet examined in the literature is negative interpretation bias. Interpretation bias refers to the tendency to interpret ambiguous or neutral information as negative or threatening [21]. Having a strong interpretation bias can lead individuals to frequently overestimate the presence of threat in daily life, experience excessive negative affect, avoid or withdraw from fulfilling activities in daily life, and experience mental health conditions such as anxiety and depression [22]. Interpretation bias is theoretically a good candidate for mediating the association between mindfulness and reduced trauma vulnerability as it has been associated with both constructs in the literature.

For example, interpretation bias has been implicated in the development and maintenance of mental health conditions associated with traumatic experiences such as PTSD, depression, and anxiety [23–25]. Most notably, Ehlers and Clarks’ [25] comprehensive and influential cognitive model of PTSD theorises that PTSD develops in those who interpret potentially traumatic events and their initial reactions to these events, such as intrusions and negative emotions, as signs of ongoing threat rather than time-limited events that no longer pose a threat in the present moment. The model theorises that people experience this ongoing sense of current threat in the form of intrusions, hyperarousal, and high emotionality long after the initial trauma, and attempt to reduce these experiences by doing things such as avoiding stimuli that might trigger memory of the trauma, ruminating over the trauma to avoid future traumas, and suppressing thoughts and emotions linked to the trauma. It is theorised that these control strategies ironically exacerbate PTSD symptoms by preventing individuals from re-appraising the meaning of their trauma experience.

This theoretical involvement of interpretation bias in PTSD is supported by empirical evidence. Most research in this area has examined the explicit negative post-trauma cognitions that can result from a threatening interpretation of the trauma and its sequelae, such as ‘the world is a dangerous place’ or ‘I have permanently changed for the worse’, and has documented a positive association between these cognitions and PTSD symptomatology and severity [26–32]. While fewer studies have examined interpretation bias directly, these is some evidence of its involvement in PTSD. One study using a trauma-related sentence completion task among a sample of combat veterans found evidence of interpretation bias being higher in those who had a diagnosis of PTSD compared to those who did not [33]. Another study of a trauma-exposed sample found that those with a diagnosis of PTSD displayed a delayed response when required to inhibit threat interpretations of homographs, consistent with interpretation bias, compared to those who did not have a diagnosis of PTSD [34]. Another study showed a sample of trauma-exposed participants incomplete sentences on a computer screen that were then completed with words that caused the meaning of the sentence to be either neutral, threatening, or non-sensible [35]. The researchers asked participants to report whether they found the sentences sensible and collected electroencephalography (EEG) data to reveal to what degree different endings were consistent or inconsistent with participants’ expectations. Participants with PTSD were more likely than those without PTSD to expect threatening sentence endings and consider them sensible.

Interpretation bias is also theoretically linked to mindfulness, given mindfulness involves applying a nonjudgmental lens to one’s present moment experience and is theorised to produce more veridical perceptions of external and internal events that are less influenced by prior beliefs and learned associations [36–38]. Though empirical research into the potential links between mindfulness and interpretation bias is still in its infancy, studies have demonstrated a negative association between mindfulness and interpretation bias (e.g., [39–41]). Two of these studies also examined the extent to which interpretation bias might mediate the association between trait mindfulness and symptoms of psychopathology. Mayer et al. [41] found that interpretation bias partially mediated a negative association between trait mindfulness and measures of anxiety and depression. Specifically, they found that participants who scored lower on trait mindfulness tended to report higher levels of anxiety and depression and that this tendency was partly explained by these participants displaying stronger interpretation bias. While promising, this pattern of results has not been found consistently. Hoge et al. [39] measured the association between trait mindfulness, interpretation bias and symptoms of Generalised Anxiety Disorder (GAD) before and after an eight-week Mindfulness-based Stress Reduction intervention. Contrary to Mayer et al. [41], Hoge et al. [39] did not find an association between baseline levels of trait mindfulness and interpretation bias. Additionally, although the mindfulness intervention increased levels of trait mindfulness, decreased interpretation bias, and decreased symptoms of GAD, they did not find evidence of interpretation bias mediating the association between changes in trait mindfulness and symptoms of GAD. These mixed results emphasize the need to clarify the potential relationships not only between mindfulness and interpretation bias, but also between these constructs and different forms of psychopathology.

The present study aimed to investigate the associations between trait mindfulness, interpretation bias, and PTSD symptomatology in a trauma-exposed sample. It was hypothesised that interpretation bias would mediate a negative association between trait mindfulness and symptoms of post-trauma psychopathology. Trait mindfulness, interpretation bias and symptoms of PTSD were assessed cross-sectionally in a sample of adults who had been exposed to at least one potentially traumatic event in their lifetime. A mediation model was predicted such that higher levels of trait mindfulness would be associated with lower PTSD symptom severity, and interpretation bias would at least partially explain this association.

## Method

### Participants

Participants were 133 undergraduate students who reported having been exposed to at least one potentially traumatic event on a self-report questionnaire assessing lifetime exposure to potentially traumatic events. The sample consisted of 103 female (77%), 29 male (22%) and 1 non-binary (1%) students with a mean age of 20.54 years (*SD* = 4.04, range 18–48). Most participants (68%) had been exposed to more than one type of potentially traumatic event and the mean number of types of potentially traumatic events participants had been exposed to was 2.59 (*SD* = 1.74, range 1–8). The mean number of years that had passed since participants’ self-rated “most troublesome” potentially traumatic event, was 6.3 years (*SD* = 6.05, range < 12 months – 29 years).

### Measures

The Traumatic Events Questionnaire (TEQ) [42] measured lifetime exposure to potentially traumatic events. The TEQ is an eleven item self-report questionnaire that assesses lifetime exposure to potentially traumatic events including both interpersonal events, such as being a victim of physical or sexual abuse, and non-interpersonal events, such as experiencing a natural disaster or car accident. Participants are asked to answer “yes” or “no” to indicate whether they have been exposed to each of the potentially traumatic events. The last two items on the TEQ give respondents the opportunity to report any other potentially traumatic events that are not specified in the questionnaire or that they “feel they can’t tell about”. The test–retest reliability of the TEQ for specific events has been shown to range from *r* = 0.72 to *r* = 1.00 [42].

The Post-Traumatic Stress Disorder Checklist – Civilian version (PCL-C) [43, 44] measured PTSD symptoms. The PCL-C is a self-report scale comprised of 17 items that assess symptoms of PTSD specified in the Diagnostic & Statistical Manual of Mental Disorders – 4^th^ Edition (DSM-IV). We used the PCL-C to allow direct comparison with previous research [24], and it also has high concordance with the newer PCL-5 (e.g. [45]). Respondents rate how much they have been bothered by each symptom on the PCL-C in the past month on a five-point Likert scale (1 = “not at all”, 5 = “extremely”). Scores on all items are summed to calculate a total symptom severity score (range = 17–85), where higher scores indicate greater symptom severity. The scale demonstrates strong test–retest reliability, internal consistency, convergent validity, and diagnostic utility [44, 46]. The PCL-C demonstrated very good reliability in the present study (Cronbach’s alpha = 0.93).

The Five Facet Mindfulness Questionnaire – Short Form (FFMQ-SF) [47] measured trait mindfulness. The FFMQ-SF is a 24-item self-report questionnaire that examines five core facets of mindfulness including observing, describing, acting with awareness, non-judging, and non-reactivity to inner experience. Participants rate how frequently or infrequently they have had each experience listed in the FFMQ-SF, in the last month, on a five-point scale (1 = “never or very rarely”, 5 = “very often or always true”). A total FFMQ-SF score is calculated (range = 24–120), where higher scores indicate higher trait mindfulness. The FFMQ-SF has demonstrated good internal consistency as well as strong convergent, discriminant and criterion validity in a sample of Dutch adults with clinically relevant symptoms of depression and anxiety [47]. The original 39-item FFMQ has also been validated in a sample of Australian adults [48]. The FFMQ-SF demonstrated good reliability in the present study (Cronbach’s alpha = 0.79).

The interpretation bias recognition task (IBRT; [49]) was used to measure general negative interpretation bias. The task consisted of eight general ambiguous threat scenarios that have been used in previous studies (e.g., [50]). These scenarios, shown in Supplementary File 1, featured a range of everyday hypothetical situations that had the potential to be interpreted as threatening to physical and psychological wellbeing. Interpretation of general rather trauma-specific scenarios was assessed for two reasons. Firstly, this allowed a broad and diverse trauma-exposed sample to be examined. Secondly, this enabled the study to investigate whether it is a general capacity to view life experiences through a nonjudgmental lens that protects naturally more mindful individuals from developing PTSD following exposure to potentially traumatic events. It was theorised that it is this general capacity, that theoretically precedes an individual’s experience of potentially traumatic events, that makes them less likely to interpret these events and their sequalae in threatening ways, and thereby less likely to develop PTSD.

At the start of the IBRT, participants are informed that they will next complete a word completion task that requires them to read short scenarios on screen, complete an incomplete word at the end of the last sentence in each scenario, and answer a comprehension question about each scenario to check their understanding. A scenario is then shown on screen with an incomplete word at the end of the final sentence (e.g. You decide that you must start to exercise more. For the next week you take a little more exercise each day. After several weeks, you are running further and decide to see how far you can push yourself, when you notice your breathing is "la-our–"). After pressing spacebar, participants are presented with a text box into which they type the word (e.g. “laboured”). After completing the incomplete word, participants are then asked a comprehension question about the scenario to ensure they have read the scenario (e.g., “Have you been exercising for several weeks?"). After completing the word fragments and comprehension questions for all eight randomly presented scenarios, participants are then presented with instructions for a surprise memory task. Specifically, participants are informed that they will be presented with four sentences describing the scenarios they had read in the previous task and asked to indicate how similar in meaning the sentences were to the original scenario. Four interpretations of each scenario are then shown together on screen, along with the scenario title. Both the scenarios in the task and the four interpretations for each scenario are presented in random order. These include a benign interpretation (e.g. “Running further than usual you have to breathe harder and deeper”), a negative or threatening interpretation disambiguation (e.g. “Pushing yourself too hard you cannot get enough air and feel dizzy”), a positive foil (e.g. “Pushing yourself more than usual you feel your running is much easier”), and a negative foil (e.g. “You push yourself so hard you strain a muscle and hurt yourself”). Participants rate how similar they think each is to the original scenario on 4-point Likert scales (1 = “very dissimilar”, 4 = “very similar”). See Supplementary File 2 for screenshots of the interpretation bias task instructions.

An interpretation bias index is computed by subtracting the mean of similarity ratings for the benign interpretations from the mean of similarity ratings for the negative interpretations. Higher index scores indicate a greater tendency to negatively interpret ambiguous scenarios, i.e., greater interpretation bias. The reliability of the similarity ratings for each of the four interpretations in the IBRT ranged between poor and good (Cronbach's alpha = 0.59, 0.55, 0.64, and 0.69, for benign interpretation, negative interpretation, positive foil, negative foil, respectively).

### Procedure

Data were collected through a one-hour web-based study using the online research software Inquisit Web (Millisecond Software, Seattle, WA, USA). Participants were recruited to the study via the University of Sydney Psychology Research Participation System and received course credit for their participation. Participants completed a battery of questionnaires and cognitive measures as part of a larger project conducted under the Cognition and Emotion Research Collaboration Initiative (CERCI). The study was conducted under reciprocal ethics approval granted by the University of Western Australia Human Research Ethics board (RA/4/1/5243). Informed consent to participate in the study was obtained in online written form from all individual participants.

Participants first completed demographic questions about characteristics such as age and gender, before filling in the TEQ. Participants were then asked to identify the traumatic event they reported on the TEQ using one or two words. If they had reported more than one event on the TEQ, participants were asked to choose the one that was “most troublesome” to them at the current time. Participants then completed the PCL-C in which they were instructed to read “a list of problems and complaints that people sometimes have in response to stressful life experiences” and rate how much they had been “bothered by” each problem or complaint in the past month. Participants were instructed to think about the event they had identified in their previous answer when filling in the PCL-C, i.e., the single event they had selected on the TEQ or the “most troublesome” event out of those they had selected on the TEQ. Participants then completed the FFMQ-SF before the IBRT.

### Data analyses

Data were inspected for normality, linearity, homoscedasticity, and influential cases. In line with Mayer et al. [41], influentiality was assessed with the criterion of Cook’s distance greater than 1 (e.g., [51]), where none of the cases exceeded the threshold (i.e., maximum = 0.25). Final data is available in Supplementary File 3. Correlations and independent samples t-tests were calculated to assess associations between the primary study variables and covariates. A simple mediation analysis using ordinary least squares path analysis was then computed using the PROCESS macro [52] in IBM SPSS Statistics 26 to test the hypothesis that interpretation bias mediates the negative relationship between trait mindfulness and symptoms of PTSD. Gender, age, exposure to interpersonal trauma and number of potentially traumatic events exposed to were included as covariates in the model as these are identified risk factors for developing PTSD following exposure to a potentially traumatic event [4, 53–55]. Interpersonal trauma items on the TEQ included being the victim of a violent crime such as rape, robbery, or assault, being the victim of child physical or sexual abuse, experiencing unwanted sexual experiences that involved the threat or use of force as an adult, and being subject to physical or other abuse in a relationship as an adult.

## Results

### Descriptive statistics and associations between study variables

General descriptive statistics and correlations between primary study variables are summarised in Table 1. Results on the FFMQ-SF indicated an overall moderate level of trait mindfulness in the sample with some variation. Results on the PCL-C indicated a high degree of variability in PTSD symptom severity. A total score of 44 is considered indicative of a probable diagnosis of PTSD among non-military populations, with a clinical interview required to confirm a diagnosis [56]. Almost half the current sample (47%) scored 44 or above. Exposure to more types of potentially traumatic experiences (*r*(131) = 0.40, *p* < 0.01) and exposure to potentially traumatic interpersonal experiences (*t*(132) = -4.05, *p* < 0.001) were positively associated with post-trauma symptoms, whereas trait mindfulness was negatively associated with post-trauma symptoms (*r*(131) = -0.34, *p* < 0.01). Exposure to more potentially traumatic events was positively associated with exposure to one or more potentially traumatic interpersonal events (*t*(132) = -7.35, *p* < 0.001). This was to be expected given exposure to more potentially traumatic events increases one’s chances of having been exposed to a potentially traumatic event of an interpersonal nature. No other significant associations were observed between variables.

**Table 1:** General descriptive statistics and correlations between primary study variables

	***M***	***SD***	**Range (min, max)**	**1**	**2**
1. FFMQ-SF^a^	61.23	9.27	38, 87		
2. IB^b^	0.05	0.50	-1.75, 1.25	-.03	
3. PCL-C^c^	41.17	15.61	17, 81	-.34^**^	-.07

*N* = 133; **p < .01; ^a^Five Facet Mindfulness Questionnaire–short form; ^b^Interpretation Bias; ^c^Post Traumatic Stress Disorder Checklist – Civilian Version

### Does interpretation bias mediate the relation between trait mindfulness and PTSD symptoms?

**Fig. 1:**
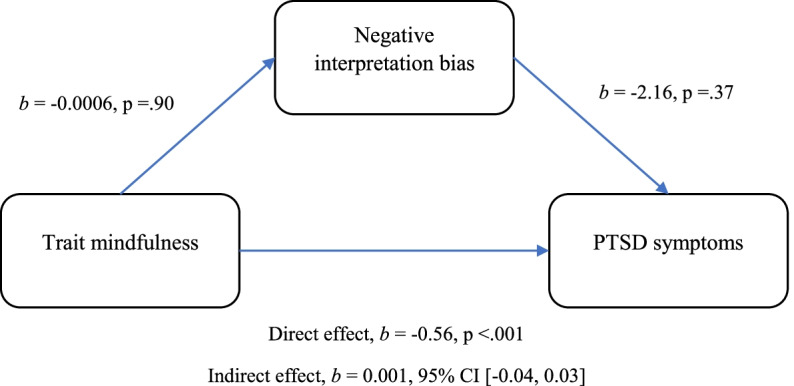
Model of trait mindfulness as a predictor of PTSD symptoms, mediated by interpretation bias. *Note:* The confidence interval for the indirect effect is a BCa bootstrapped CI based on 5000 samples

Regression coefficients of the mediation analysis are displayed in Fig. 1. Trait mindfulness, interpretation bias, and the covariates, gender, age, exposure to interpersonal trauma, and number of types of potentially traumatic events, together accounted for 29% of the variance in PTSD symptoms [*F*(6,126) = 8.68, *p* < 0.001]. There was a significant total effect of trait mindfulness on PTSD symptoms (*p* < 0.001). The indirect effect of trait mindfulness on PTSD symptoms through interpretation bias was not significant [BCa CI [-0.04, 0.03)]. Trait mindfulness was not a significant predictor of interpretation bias (*p* > 0.1) and interpretation bias was not a significant predictor of PTSD symptoms (*p* > 0.1). Among the covariates included in the model, the only construct to significantly predict PTSD symptoms was the number of types of potentially traumatic events participants had been exposed to (*b* = 2.77, *p* = 0.001). Age, gender, and exposure to an interpersonal potentially traumatic event did not significantly predict PTSD symptoms [*b* = 0.06, *p* = 0.85; *b* = 0.37, *p* = 0.90; *b* = 4.73, *p* = 0.10, respectively]. The mediation analysis was repeated a) controlling for the number of years that had passed since each participant’s exposure to the potentially traumatic event they reported for the PCL-C, and b) with all covariates removed. The effects did not change with these changes to the covariates.

## Discussion

This study aimed to investigate possible mechanisms involved in symptoms of psychopathology following a potentially traumatic event, to ultimately advance the prevention and treatment of these symptoms. It was the first to investigate whether differences in interpretation bias account for why people who are more mindful experience less severe post-trauma psychopathology. Using a cross-sectional design in a sample of undergraduate students who had experienced at least one potentially traumatic event, a significant association was found between greater trait mindfulness and lower PTSD symptom severity. This association was not, however, mediated by interpretation bias, and thus the study’s central hypothesis was not supported. The study also did not find evidence of an association between trait mindfulness and interpretation bias, nor between interpretation bias and post-trauma psychopathology.

The negative association between trait mindfulness and post-trauma symptoms is consistent with previous research suggesting that being more mindful may confer a buffer to the psychological effects of trauma [10–14]. This finding provides further support for the ongoing inclusion of mindfulness in PTSD prevention and treatment research. In the absence of significant mediation by interpretation bias, other mechanisms may better account for the link between mindfulness and reduced trauma symptoms severity. Several mechanisms have partly explained this association in prior literature. These include lower levels of cognitive fusion, that is, the tendency to identify with one’s thoughts and feelings [12], higher levels of cognitive reappraisal of the traumatic event, that is, the act of consciously changing one’s interpretation of an event to be more neutral or positive [18], lower levels of experiential avoidance, that is, the tendency to avoid experiencing uncomfortable thoughts, emotions, bodily sensations, and memories [17], and lower levels of expressive suppression, that is, the act of consciously inhibiting one’s automatic emotional expressions in an attempt to suppress uncomfortable emotions [18].

Taken together, the results of the current study and previous literature may indicate people who are more mindful show less severe post-trauma psychopathology not because they are less likely to initially interpret ambiguous or neutral information as threatening but instead because they are more likely to cognitively defuse from and challenge threatening interpretations of life experiences. Given the overall paucity of studies that have examined these mechanisms however, and the predominance of studies utilising cross-sectional designs, a greater number of studies utilising longitudinal designs are needed to confirm causal relationships between mindfulness, trauma symptoms and each of these mechanisms. Additionally, all studies conducted so far have examined only one or two mediators in any single mediation analysis, highlighting the need for further studies to examine more mediators in multiple mediator models so that the relative importance of each mechanism can be assessed.

The low and statistically non-significant association between trait mindfulness and interpretation bias found in the present study was contrary to the theoretical links between these constructs [36–38] and a prior study that documented an association between higher levels of trait mindfulness and lower levels of interpretation bias [41]. It was, however, consistent with another study that did not replicate this association [39]. The differing results of Mayer et al. [41], Hoge et al. [39], and the current study may point to a difference in the relationship between trait mindfulness and interpretation bias in different populations. Specifically, it could be that higher levels of trait mindfulness are associated with lower levels of interpretation bias in general populations, such as that sampled by Mayer et al. [41], but not in those experiencing significant mental health problems, such as that sampled by Hoge et al. [39], nor in those who have been exposed to one or more potentially traumatic events, such as that examined in the current study. Further studies comparing associations between trait mindfulness and interpretation bias in clinical, nonclinical, trauma exposed, and non-trauma exposed samples are needed to clarify the relationship between these constructs in these varying populations.

Another explanation could be that these studies produced different results due to differences in the way they each measured interpretation bias. In the only study to observe a mediating effect of interpretation bias, Mayer et al. [41] examined interpretation bias using ambiguous scenarios that were designed to assess interpretation biases evident in anxiety and depression. While some of these scenarios share similarity with those utilised in the current study (e.g., “You wake up in the middle of the night because of a loud noise. What is going through your mind?”; response options: “Probably it’s something outside or at the neighbour’s” “Oh no, there’s an intruder!”), most posed less severe threats to physical and psychological wellbeing (e.g., “You are giving a presentation and notice two persons laughing. What is going through your mind?”, response options: “They are having fun time together” “They are laughing about me”). It may be that a milder level of interpretation bias is able to be detected with this task than the IBRT used in the present study. It is however also worth noting that Mayer et al. used an assessment task that appears susceptible to demand or response bias effects, as the two response options are presented immediately after the scenario. For both the IBRT used in the present study, and the homophone priming task used by Hoge et al. [39], response options are not presented immediately after each scenario, and are therefore less likely to be susceptible to immediate response bias. However, the IBRT was presented after questionnaires in the present study, and therefore may have been susceptible to a priming effect. Given that only a single study has shown such a mediating effect, the other null fundings suggest that it will be important to replicate such a finding using a robust interpretation bias measure, and until such time this potential relationship may be regarded with caution.

The weak and statistically non-significant association between interpretation bias and PTSD symptomatology was also contrary to theoretical models of PTSD that incorporate interpretation bias as a causal and perpetuating factor [24–29, 31, 32]. It was also contrary to the results of prior studies that have documented higher levels of interpretation bias in individuals who develop PTSD following exposure to a potentially traumatic event compared to those who do not develop PTSD [33–35]. It should be noted, however, that these studies examined trauma-specific interpretation bias in individuals exposed to the same or similar trauma, whereas the current study examined general interpretation bias in a sample of individuals who had been exposed to diversely different traumas. For example, the interpretation bias sentence completion task used by Kimble et al. [33] presented combat veterans with sentences that could be completed with either non-military related words that made the sentences non-threatening or military-related words that made the sentences indicate the presence of a military combat threat. Thus, it could be that a relationship exists between trauma-specific interpretation bias and PTSD but not general negative interpretation bias and PTSD.

These studies also examined differences between groups of people who did and did not meet criteria for a diagnosis of PTSD. The current study instead examined variations in PTSD symptomatology among a non-clinical sample. Although almost half the current sample (47%) scored at or above the threshold for a probable diagnosis of PTSD, unlike in the other studies, there was no clinical interview conducted to confirm this diagnosis. Thus, it could be that there is an association between general interpretation bias and PTSD symptomatology, however this only holds in clinical samples. It should also be noted that these studies examined between group differences in relatively small samples and so require further replication among more robust samples to substantiate their findings. To further clarify the relationship between interpretation bias and trauma symptoms, it is recommended that future studies examine both trauma-specific interpretation bias and general interpretation bias among robust trauma-exposed samples that include both people with and without clinically significant PTSD symptomatology. It is possible that PTSD-specific interpretation bias may show associations with higher levels of PTSD compared to general negative interpretation. However, while some cognitive biases (e.g. biased attention) are known to be highly specific, biased interpretation is often observed to be generally negative rather than associated with condition-specific information [57, 58]. It is also recommended that these studies assess trait mindfulness in addition to different forms of interpretation bias to determine whether the link between mindfulness and trauma symptoms may be partly explained by more trauma-specific interpretation bias rather than the general interpretation bias assessed in the current study.

It should also be recognised that we cannot rule out the possibility that the present study may have failed to detect small associations between interpretation bias and the two other primary study variables, mindfulness, and PTSD symptom severity, due to being underpowered. Our sample size was, however, similar to Mayer et al.’s [41]. It is also possible that the low reliability of the IBRT may have impacted on the findings of the present study. It is important to consider how low reliability in cognitive tasks [59], such as the IBRT, may limit the ability to test constructs of interest, such as interpretation bias. Nonetheless, cognitive processes themselves can be inherently variable [60], and further work to differentiate task vs construct unreliability is essential. The IBRT has been successfully employed in prior studies where it demonstrated a similar level of reliability [50, 61]. Finally, the cross-sectional nature of the present study limits conclusions that can be drawn about directionality. Further tests of mediation over time would help to confirm whether mindfulness does buffer against trauma symptoms, as well as the mechanisms through which it may work.

## Conclusion

Improving our understanding of the mechanisms involved in buffering against trauma symptoms following a potentially traumatic event, and the treatment of post-traumatic stress symptoms is crucial for improving prevention and treatment interventions for PTSD, a condition that affects approximately 1 in 10 people exposed to potentially traumatic events. The present study was the first to assess the degree to which interpretation bias mediates the association already identified in the literature between being more mindful and showing less severe post-trauma psychopathology. The study provided further evidence of a negative association between trait mindfulness and PTSD symptoms, however did not find evidence to support the hypothesis that variation in interpretation bias partly accounts for this effect.

## Supplementary Information

**Additional file 1. **General threat scenarios and interpretations from Interpretation Bias Recognition Task.

**Additional file 2. **Screenshots of instructions for interpretation bias task.

**Additional file 3. **De-identified study data.

## Data Availability

All data generated or analysed during this study are included in a de-identified form in a supplementary information file associated with this published article.

## Abbreviations

CERCI: Cognition and Emotion Research Collaboration Initiative
DSM-IV: Diagnostic & Statistical Manual of Mental Disorders – 4th Edition
EEG: Electroencephalography
FFMQ-SF: Five Facet Mindfulness Questionnaire – Short Form
IBRT: Interpretation bias recognition task
PCL-C: Post-Traumatic Stress Disorder Checklist – Civilian version
PTSD: Post-traumatic Stress Disorder
TEQ: Traumatic Events Questionnaire
