# Supplementary material for: For there is nothing either good or bad: a study of the mediating effect of interpretation bias on the association between mindfulness and reduced post-traumatic stress vulnerability
Source: BMC Psychiatry. 2022 May 12;22:329. doi: 10.1186/s12888-022-03950-y (PMC9097341; doi:10.1186/s12888-022-03950-y)
Supplement: Supplementary file 1 — Additional file 1. General threat scenarios and interpretations from Interpretation Bias Recognition Task. [file 12888_2022_3950_MOESM1_ESM.pdf]

Supplementary File 1

General threat scenarios and interpretations from Interpretation Bias Recognition Task

Scenario	Possible solutions
You have been feeling dizzy occasionally, and decide to get a check-up. You make an appointment right away. Your doctor takes your blood pressure and listens to your chest, and then tells you to relax while giving you his opinion.	Benign interpretation: The doctor tells you that there is absolutely nothing to worry about.<br><br>Threatening interpretation: The doctor tells you to relax and gives you an opinion on your disease.<br><br>Positive foil: The doctor tells you that you have made a complete recovery.<br><br>Negative foil: The doctor tells you that you will need another course of treatment.
You are at home alone late one night. You have just finished reading and turn out the light to go to sleep. While lying in the dark you hear a soft rustling sound coming from just outside your window.	Benign interpretation: Lying in bed you hear the wind blowing something outside your window.<br><br>Threatening interpretation: Lying in the dark you hear soft footsteps from just outside your window.<br><br>Positive foil: Lying in bed you hear the sound of birds singing outside your window.<br><br>Negative foil: Lying in bed you hear a car accident in the street outside your window.
You are on your way to a holiday abroad. You have been in the air for an hour when you hear a change in the sound of the engine next to you. The fasten seat belt light flashes, and you hear the captain begin to make announcement.	Benign interpretation: The seat belt light comes on and the captain says you will be landing soon.<br><br>Threatening interpretation: The seat belt light comes on and the captain tells you one engine is failing.<br><br>Positive foil: The seat belt light is on and the cabin crew say lunch will be served soon.<br><br>Negative foil: The seat belt light is on and the cabin crew say you are diverted due to fog.

You have been offered a routine cancer screening appointment at your local health centre. You have an X-ray and some samples are taken for tests. While waiting you see the Doctor point out something on the X-ray plate to the nurse.	Benign interpretation: You see the Doctor pointing out to the nurse that your X-ray is normal.<br><br>Threatening interpretation: You notice the Doctor pointing out a tumour on your X-ray to the nurse.<br><br>Positive foil: You hear the Doctor telling the nurse to inform you everything is fine.<br><br>Negative foil: You watch the nurse taking the X-ray of your tumour to the Doctor.
You have been visiting friends in the centre of town, when you realise it is getting late. They offer you a lift but you set off on foot. Walking through a street that you don't know at all well, you can hear someone running up from behind.	Benign interpretation: In an unfamiliar street you hear a jogger running up and past you.<br><br>Threatening interpretation: In a street you don't know well you hear a mugger running up behind you.<br><br>Positive foil: In a street you don't know you meet an old friend who walks home with you.<br><br>Negative foil: In an unfamiliar street someone steps out and threatens you with a knife.
You are finding that your sight is less good than it was, and decide to try the laser surgery you have read about. After the operation your eyes are still bandaged, and you realise that your life will be affected radically by the result.	Benign interpretation: You realise that this operation is likely to improve your life a great deal.<br><br>Threatening interpretation: You realise how awful your life will be if the operation is a failure.<br><br>Positive foil: You think how pleased you will be with the improvement in your looks.<br><br>Negative foil: You think that you had not realised how painful the surgery would be.

You are preparing for the removal men, and packing your things in boxes. You finish a large box and try lifting it to one side. As you lift it you feel a pain in your chest and down your arm and let the box drop onto the floor.	Benign interpretation: You feel a pain and decide that you are not strong enough to lift the box.<br><br>Threatening interpretation: You feel a pain that could mean you are straining your heart.<br><br>Positive foil: You feel a pain and decide to let the removal men take over.<br><br>Negative foil: Your damaged back gives out and you let go of the box right away.
You decide that you must start to exercise more. For the next week you take a little more exercise each day. After several weeks, you are running further and decide to see how far you can push yourself, when you notice your breathing is laboured.	Benign interpretation: Running further than usual you have to breathe harder and deeper.<br><br>Threatening interpretation: Pushing yourself too hard you cannot get enough air and feel dizzy.<br><br>Positive foil: Pushing yourself more than usual you feel your running is much easier.<br><br>Negative foil: You push yourself so hard you strain a muscle and hurt yourself.
