# Supplementary material for: For there is nothing either good or bad: a study of the mediating effect of interpretation bias on the association between mindfulness and reduced post-traumatic stress vulnerability
Source: BMC Psychiatry. 2022 May 12;22:329. doi: 10.1186/s12888-022-03950-y (PMC9097341; doi:10.1186/s12888-022-03950-y)
Supplement: Supplementary file 2 — Additional file 2. Screenshots of instructions for interpretation bias task. [file 12888_2022_3950_MOESM2_ESM.pdf]

Supplementary File 2

Screenshots of instructions for interpretation bias task

The next task is a word completion task.
You will read short scenarios on the screen, and at the end of each scenario is a word that has some missing letters.

An example of such a scenario is:

"You decide to go to the movies with your friends.
You get to the theater and there is a long line.
Because of the line, you are
l-te."

Your task is to complete the word fragment (in the example: "late"). Use your understanding of the scenario to complete the word.
After each scenario, a question will be asked to check your understanding of the scenario. These will always be yes/no questions.
For example, a question related to the above scenario can be: "Did you make it to the movie on time?" In this case, the answer is "no".

Press the spacebar to continue with the instructions.

Each scenario will be presented to you one line at a time, ending with the last word. As soon as you know what the missing letters in the word are, press the SPACEBAR.
On the next screen, you'll be able to type in the full word (using the keyboard).
Once you're done with this, press ENTER to go to the question.
Use the mouse to answer the question by clicking on your response.
You'll get feedback on whether or not your answer is correct before you move on to the next scenario.

Thus, all you have to do is read the scenario, press the SPACEBAR when you know what the missing letters in the last word are, and type the word.
Next, you answer the question using the mouse.

Press the spacebar to start.

The exercise regime

You decide that you must start to exercise more. For the next week you take a little more exercise each day. After several weeks, you are running further and decide to see how far you can push yourself, when you notice your breathing is la-our--

This next task will test your memory for some of the scenarios.

You'll see 4 different sentences that each describe one of the scenarios you have seen before.
At the top of the page is a little hint to the content of the scenario.
For each of these sentences, we ask you to indicate how similar in meaning this sentence is to the scenario that was presented before.
You'll rate this similarity on a scale from 'very dissimilar' to 'very similar'.

The ratings of these 4 sentences are independent from one another, so you don't have to pick a different answer for each of them. It is possible for example, that you find that 2 of the 4 sentences are 'very similar' to a scenario described before.
Just rate the similarity of each sentence on its own, without taking into account the other sentences.

Press the spacebar to start.
